# Liability of Hospitals in Malpractice Suits

**Published:** 1880-06

**Authors:** 


### LIABILITY OF HOSPITALS IN MALPRACTICE SUITS.

THE Supreme Court of Rhode Island, according to the *Maryland Medical Journal*, December, 1879, has recently decided that hospital corporations should be considered liable for failure to exercise reasonable care in selecting skillful, competent men as internes. This decision grows out of a case where suit for malpractice was instituted against a Rhode Island Charity Hospital, by a patient whose fingers were cut off by a circular saw. Hemorrhage was excessive, and was only controlled by the use of the tourniquet, which instrument was kept on for seventeen hours. The result was the arm was amputated at the shoulder joint, the patient affirming that careless treatment upon the part of the interne, had induced the result. The court directed the jury to give a verdict for the defendant, on the ground that a charity institution should not be made liable for negligence or unskillful treatment. The case was taken to the Supreme Court, with the above decision.—*Medical News.*

